# Intrathyroidal parathyroid carcinoma in a patient with multiple endocrine neoplasia type 1

**DOI:** 10.1210/jcemcr/luag211

**Published:** 2026-07-29

**Authors:** Arita Thatte, Marina Basina, Kaniksha Desai

**Affiliations:** Division of Endocrinology, Gerontology, and Metabolism, Department of Medicine, Stanford University School of Medicine, Stanford, CA 94305, USA; Division of Endocrinology, Gerontology, and Metabolism, Department of Medicine, Stanford University School of Medicine, Stanford, CA 94305, USA; Division of Endocrinology, Gerontology, and Metabolism, Department of Medicine, Stanford University School of Medicine, Stanford, CA 94305, USA

**Keywords:** parathyroid carcinoma, thyroid nodule, intrathyroidal parathyroid, MEN1 syndrome

## Abstract

Primary hyperparathyroidism occurs in up to 95% of patients with multiple endocrine neoplasia type 1 (MEN1). However, only a few cases of parathyroid carcinoma have been reported, and only one case of intrathyroidal parathyroid carcinoma. We describe the case of a 41-year-old male with a history of MEN1 and primary hyperparathyroidism who developed hypercalcemia to 13.1 mg/dL (SI: 3.3 mmol/L) (reference range [RR], 8.6-10.3 mg/dL [SI: 2.2-2.6 mmol/L]) with parathyroid hormone (PTH) 454 pg/mL (SI: 48.1 pmol/L) (RR, 15-65 pg/mL [SI: 1.6-6.9 pmol/L]). Neck ultrasonography showed a highly suspicious intrathyroidal 3.6 cm nodule. The patient had a lobectomy, and final pathology showed a 3.1 × 1.6 × 1.2 cm parathyroid carcinoma. After the surgery, calcium dropped to 6.0 mg/dL (SI: 1.5 mmol/L) with PTH of 11 pg/mL (SI: 1.2 pmol/L) requiring oral calcium supplements, calcitriol, and hydrochlorothiazide. Laboratory tests at 12 months on these medications showed calcium of 9.3 mg/dL (SI: 2.3 mmol/L) and PTH 21 pg/mL (SI: 2.2 pmol/L) indicating recovery of parathyroid function without recurrence, and calcitriol was discontinued. This case illustrates that a high index of suspicion for malignancy should be maintained for intrathyroidal parathyroid adenoma in the setting of severe hypercalcemia, especially in MEN1.

## Introduction

Multiple endocrine neoplasia type 1 (MEN1) is an autosomal dominant genetic disorder caused by a germline pathogenic variant in the *MEN1* tumor suppressor gene. Benign parathyroid disease occurs in greater than 95% of patients [1]; however, parathyroid carcinoma is extremely rare; fewer than 20 cases of have been reported [2]. Intrathyroidal location is the rarest ectopic site for parathyroid glands, accounting for about 0.2% of cases [3]. We describe a case of intrathyroidal parathyroid carcinoma in a patient with MEN1, with a known inactivating pathogenic variant of the *MEN1* gene, and the challenges associated with diagnosis.

## Case presentation

A 41-year-old male with a known *MEN1* inactivating pathogenic variant c.563_564delCC (p.Pro188Glnfs*7), previously treated hyperparathyroidism was found to have hypercalcemia during hospitalization after Whipple resection for pancreatic neuroendocrine tumor (NET). His past surgical history included prior parathyroid surgery at age 20 for hypercalcemia when the right and left inferior parathyroid glands were excised, the other glands could not be identified. After intermittent care due to access issues, he re-presented at the age of 35 with serum calcium of 12.3 mg/dL (SI: 3.1 mmol/L) (reference range [RR], 8.6-10.3 mg/dL [SI: 2.2-2.6 mmol/L]) and parathyroid hormone (PTH) of 158 pg/mL (16.7 pmol/L) (RR, 15-65 pg/mL [SI: 1.6-6.9 pmol/L]) in the setting of recurrence of hyperparathyroidism.

He underwent resection of the right superior parathyroid gland, and a subtotal resection of the left inferior parathyroid gland with improvement in calcium levels to 10.7 mg/dL (SI: 2.7 mmol/L) with PTH of 41 pg/mL (SI: 4.3 pmol/L). Other manifestations of MEN1 included pancreatic and small bowel NET diagnosed at age 33 in the context of gastroesophageal reflux disease, with metastatic disease to the stomach and lungs discovered on positron emission tomography (PET) scan at age 41 after a lapse of care. His only medication was omeprazole 40 mg twice daily. Family history was notable for MEN1 in his father, brother, paternal grandmother, and paternal aunt, all of whom had a history of benign parathyroid adenomas.

## Diagnostic assessment

Hypercalcemia was discovered incidentally during routine laboratory testing on hospital admission. The patient denied any symptoms of hypocalcemia. Neck exam showed healed linear scars from prior parathyroid surgeries and no palpable neck mass. Laboratory workup revealed serum calcium of 13.1 mg/dL (SI: 3.3 mmol/L) corrected for albumin was 13.6 mg/dL (SI: 3.4 mmol/L).

Additional workup showed a PTH of 454 pg/mL (SI: 48.1 pmol/L) and a low vitamin D (25-hydroxy) level of 8 ng/mL (SI: 20 nmol/L) (RR, 30-100 ng/mL [SI: 75-250 nmol/L]). Vitamin D (1,25 dihydroxy) was normal at 26 pg/mL (SI: 62 pmol/L) (RR, 18-64 pg/mL [SI: 43-154 pmol/L]). Parathyroid hormone-related peptide (PTHrP) level was undetectable. Serum creatinine was 0.9 mg/dL (SI: 78 µmol/L) (RR, 0.6-1.3 mg/dL [SI: 53-114 µmol/L]). See Table 1 for a summary of laboratory parameters.

**Table 1:** Serum calcium and other relevant lab parameters before and after surgery

Lab value	Reference range	Labs during initial hospitalization	Labs 2 weeks after surgery*^a^*	Most recent labs*^b,c^*
Calcium, serum	8.6-10.3 mg/dL (2.2-2.6 mmol/L)	**13.1 mg/dL** **(3.3 mmol/L)**	**6.0 mg/dL** **(1.5 mmol/L)**	8.6 mg/dL (2.2 mmol/L)
Albumin, serum	3.6-5.1 g/dL (36-51 g/L)	**3.4 g/dL** **(34 g/L)**	**3.1 g/dL** **(31 g/L)**	4.3 g/dL (43 g/L)
Calcium (corrected for albumin)	8.6-10.3 mg/dL (2.2-2.6 mmol/L)	**13.6 mg/dL** **(3.4 mmol/L)**	**6.7 mg/dL** **(1.7 mmol/L)**	**8.4 mg/dL** **(2.1 mmol/L)**
Phosphorus, serum	2.5-4.5 mg/dL (0.8-1.5 mmol/L)	**2.2 mg/dL** **(0.7 mmol/L)**	**5.4 mg/dL** **(1.7 mmol/L)**	3.0 mg/dL (1.0 mmol/L)
Creatinine, serum	0.6-1.3 mg/dL (53-114 µmol/L)	0.9 mg/dL (78 µmol/L)	1.0 mg/dL (88 µmol/L)	1.1 mg/dL (101 µmol/L)
Parathyroid hormone	15-65 pg/mL (1.6-6.9 pmol/L)	**454 pg/mL** **(48.1 pmol/L)**	**11 pg/mL** **(1.2 pmol/L)**	28 pg/mL (3.0 pmol/L)
PTHrP	≤39.6 pg/mL (≤4.2 pmol/L)	<3.8 pg/mL (<0.4 pmol/L)	ND	ND
25-hydroxy vitamin D	30-100 ng/mL (75-250 nmol/L)	8 ng/mL (20 nmol/L)	ND	55 ng/mL (137 nmol/L)
1,25-dihydroxy vitamin D	18-64 pg/mL (43-154 pmol/L)	26 pg/mL (62 pmol/L)	ND	ND

Abnormal values are shown in bold font.

Abbreviations: ND, no data; PTHrP, parathyroid hormone-related peptide.

^*a*^Two weeks after parathyroid carcinoma resection.

^*b*^Thirteen months after parathyroid carcinoma resection.

^*c*^On calcium and hydrochlorothiazide.

**Figure 1:**
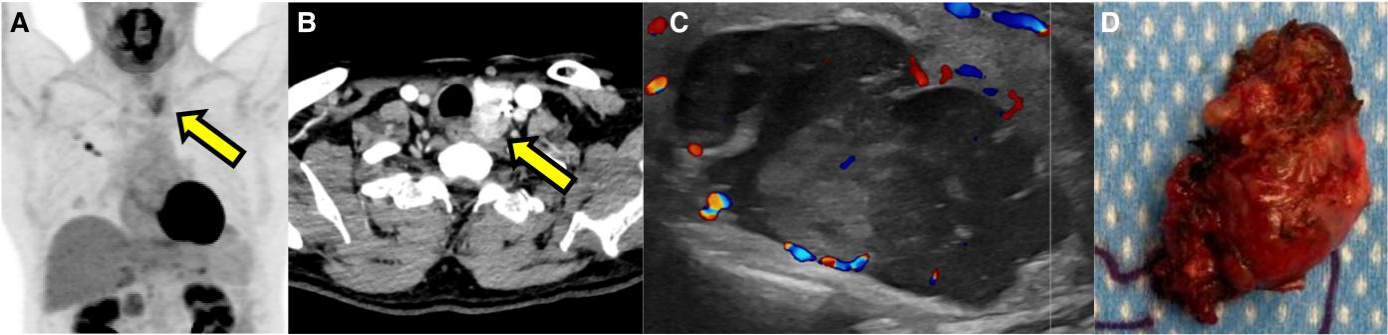
Parathyroid carcinoma imaging and pathology. (A) Fluorodeoxyglucose (FDG) positron emission tomography—computed tomography (PET-CT) showed presumed thyroid nodule (arrow) with increased FDG avidity. (B) CT scan showed left neck mass (arrow) posterior to the left thyroid bed. (C) Thyroid ultrasound showed 3.6 cm nodule (arrow) which appeared intrathyroidal. (D) Resected intrathyroidal parathyroid carcinoma gross pathological specimen.

Upon review of prior imaging, fluorodeoxyglucose (FDG) PET scan done a few months prior to admission showed a nodule in the left posterior thyroid bed with mild focal uptake, standardized uptake value of 3.9. Given the degree of PTH elevation, computed tomography (CT) scan of the neck was obtained, which showed a 2.8 cm mass posterior to the left thyroid bed. No parathyroid adenoma was noted. Neck ultrasonography showed a highly suspicious 3.6 cm solid, hypoechoic left thyroid nodule with irregular margins and possible extrathyroidal extension. There was a hyperechoic line on the ventral surface of the nodule. Minimal blood flow and no feeding vessels were seen. (Fig. 1A-C). Because the nodule appeared highly suspicious for thyroid cancer, fine-needle aspiration (FNA) biopsy was performed without PTH washout. Cytology showed a follicular lesion of undetermined significance. Given the degree of hypercalcemia and the concern for ectopic parathyroid tissue, molecular testing was obtained through Thyroseq v3 Genomic Classifier and showed high expression of chromogranin A and low but distinct expression of PTH. In the setting of MEN1, the sample was classified as likely parathyroid in origin. Prior genetic testing was negative for a germline *CDC73, RET,* or *CASR* pathogenic variant.

## Treatment

For hypercalcemia with a corrected calcium level of 13.6 mg/dL (SI: 3.4 mmol/L), the patient was initially treated with normal saline 150 ccs/h. Severe vitamin D deficiency of 8 ng/mL (SI: 20 nmol/L) was treated with ergocalciferol 50 000 IU twice weekly. Cinacalcet 30 mg twice daily was started before a bisphosphonate while initiating vitamin D replacement to avoid hypocalcemia. After 2 doses of ergocalciferol, 4 mg of zoledronic acid was administered, with serum calcium improvement to 9.7 mg/dL (SI: 2.4 mmol/L), albumin-corrected 10.3 mg/dL (SI: 2.6 mmol/L). Endocrine surgery was consulted, but given stabilization of calcium, surgery was deferred to the outpatient setting. The patient was discharged on a home regimen of cinacalcet 30 mg twice daily with a maintenance dose of cholecalciferol 5000 IU daily.

After discharge, a partial left thyroid lobectomy was performed with removal of the intrathyroidal parathyroid gland. Pathology showed a 3.1 cm × 1.6 cm × 1.2 cm parathyroid carcinoma with capsular invasion into surrounding thyroid tissue and focal perineural invasion abutting the resection margin (Fig. 1D).

## Outcome and follow-up

Post-operatively, the patient's ionized calcium level was 5.1 mg/dL (SI: 1.27 mmol/L) (RR 4.5-5.3 mg/dL [SI: 1.12-1.32 mmol/L]). He was discharged on elemental calcium 1500 mg twice daily and calcitriol 0.25 mcg daily but did not start these medications due to lack of insurance coverage. He was subsequently admitted to the hospital with symptoms of paresthesia, palpitations and dizziness, and found to have a calcium of 6.0 mg/dL (SI: 1.5 mmol/L), albumin-corrected of 6.7 mg/dL (SI: 1.7 mmol/L) with a PTH of 11 pg/mL (SI: 1.2 pmol/L). He was treated with IV calcium. Once albumin-corrected calcium improved to 7.8 mg/dL (SI: 2.0 mmol/L) and the symptoms resolved, the patient was discharged on elemental calcium 800 mg daily and calcitriol 0.25 mcg twice daily. Vitamin D3 5000 IU daily was continued.

Hypoparathyroidism persisted at subsequent follow-up visits. One month after surgery he developed nephrolithiasis after which hydrochlorothiazide 25 mg daily was started. A 24-hour urine calcium collection was ordered but not completed by the patient. In the first 12 months after surgery, the regimen was adjusted to elemental calcium 400-800 mg daily, hydrochlorothiazide 12.5-25 mg daily, and calcitriol 0.25-0.5 mcg daily for calcium (corrected for albumin) goal range of 8.0-8.5 mg/dL (SI: 2.0-2.1 mmol/L). At 12-month follow-up, labs showed calcium level of 9.3 mg/dL (SI: 2.3 mmol/L), albumin-corrected of 9.0 mg/dL (SI: 2.3 mmol/L), and PTH 21 pg/mL (SI: 2.2 pmol/L) suggesting recovery of parathyroid function without evidence of recurrence, after which calcitriol was discontinued. Ultrasonography of the neck done 15 months after carcinoma resection did not show any evidence of recurrence. Subsequently, calcium level remained in the desired range at 8.6 mg/dL (SI: 2.2 mmol/L), with albumin-corrected calcium of 8.4 mg/dL (SI: 2.1 mmol/L).

## Discussion

Parathyroid carcinoma accounts for only about 1% of all primary hyperparathyroidism cases [4]. The incidence of intrathyroidal parathyroid adenomas ranges from 0.7% to 6% [5]. The exact prevalence of intrathyroidal parathyroidal carcinoma is unknown. Benign parathyroid disease affects >95% of patients with MEN1 [1], but parathyroid carcinoma is rare. To our knowledge, this is the second published case of intrathyroidal parathyroid carcinoma in a patient with MEN1 [6].

The classic triad for parathyroid cancer consists of PTH greater than 3-4 times the upper limit of normal, serum calcium greater than 14 mg/dL (SI: >3.5 mmol/L), and palpable neck mass. Neck mass may be absent in 25% of cases, as in our patient [7].

Diagnosis can be challenging when a suspicious, intrathyroidal nodule and hypercalcemia co-exist. FNA biopsy, the main diagnostic tool, has similar appearance of thyroid and parathyroid tissue on cytology [8]. PET avidity of the nodule raised suspicion for malignancy, regardless of origin.

Parathyroid origin was confirmed by molecular testing with Thyroseq v3 Genomic Classifier on an FNA sample showing high expression of chromogranin A, and low but distinct expression of PTH. PTH washout can also help distinguish parathyroid vs thyroid tissue from an FNA biopsy [9].

FNA biopsy is not recommended if the nodule is known to be parathyroid due to the risk of malignant seeding or parathyromatosis [10]. Certain echocardiographic features can help to differentiate thyroid from parathyroid nodules, particularly the hyperechoic line on the ventral surface of the nodule, which was present in this case, and feeding vessels, which were not [11]. In this case, FNA was pursued due to lack of clarity from imaging findings alone and the need to rule out thyroid malignancy. If parathyroid adenoma is suspected and biopsy is performed for diagnosis or surgical planning, PTH washout should be performed.

In addition to *MEN1*, parathyroid carcinoma is associated with *CDC73, RET,* and *CASR* germline pathogenic variants, all of which were negative in our patient [12].

Recurrence rates may be as high as 50-80% at 10 years, so lifelong monitoring with calcium and PTH levels is required, some sources also recommend periodic neck ultrasonography [13] [14]. Parathyroid carcinoma has a moderate prognosis with estimated 5- and 10-year survival rates between 78% and 85% and between 49% and 70%, respectively. Morbidity generally occurs due to hypercalcemia [15].

In our case, the recurrence was ruled out by persistent post-operative hypoparathyroidism. Transient hypocalcemia after parathyroidectomy is common, but most cases resolve by 6 months [16]. The longer duration in our case may have been due to the history of prior neck surgeries and low vitamin D level at the time of presentation as well as the high preoperative levels of calcium and PTH.

This case highlights that a high index of suspicion should be maintained for intrathyroidal parathyroid adenomas mimicking thyroid nodules in patients with severe hypercalcemia and significantly elevated PTH, especially in the setting of MEN1 when no enlarged parathyroid gland is identified.

## Learning points

- Parathyroid carcinoma is a rare manifestation of MEN1 and should be considered in patients with severe biochemical hyperparathyroidism.
- Marked hypercalcemia and significantly elevated PTH levels may suggest parathyroid carcinoma, even in the absence of a palpable neck mass.
- Intrathyroidal parathyroid carcinoma may mimic a thyroid nodule, and tissue origin can be difficult to determine with FNA cytology alone; molecular testing can help confirm parathyroid origin and PTH washout should be considered at the time of the FNA.
- Medical therapy (eg, cinacalcet, bisphosphonates) can control hypercalcemia as a bridge to surgery, with long-term surveillance using serum calcium and PTH due to high risk of recurrence.

## Contributors

All authors made individual contributions to authorship. A.T., K.D., and M.B. were involved in diagnosis and management of the patient. All authors edited, reviewed, and approved the final draft.

## Data Availability

Data sharing is not applicable to this article as no datasets were generated or analyzed during the current study.
